# Resting Stroop task: Evidence of task conflict in trials with no required response

**DOI:** 10.3758/s13423-023-02354-7

**Published:** 2023-08-24

**Authors:** Ronen Hershman, Gal Dadon, Andrea Kiesel, Avishai Henik

**Affiliations:** 1 Department of Psychology and The Zelman Center for Brain Science, Ben-Gurion University of the Negev, Beer-Sheva, Israel; 2 Department of Psychology, Innsbruck University, Innrain 52f, Innsbruck, 6020 Austria; 3 Department of Psychology, University of Freiburg, Freiburg, Germany

**Keywords:** Task conflict, Informational conflict, Stroop effect, Pupillometry, Cognitive control

## Abstract

In the typical Stroop task, participants are presented with color words written in different ink colors and are asked to respond to their color. It has been suggested that the Stroop task consists of two main conflicts: information conflict (color vs. word naming) and task conflict (respond to color vs. read the word). In the current study, we developed a novel task that includes both Response trials (i.e., trials in which a response is required) and Rest trials (i.e., trials in which no response is required or available) and investigated the existence of both information and task conflicts in Rest trials. We found evidence for task conflict in Response and also in Rest trials, while evidence for information conflict was only observed in Response trials. These results are in line with a model of task conflict that occurs independently of and prior to information conflict in the Stroop task.

The Stroop task (Stroop, 1935) has been frequently used to examine cognitive control. Specifically, it has been employed to examine the ability to focus on relevant information while ignoring irrelevant information. In the commonly used Stroop task (Henik et al., 2018), participants are presented with a color word printed in color and are asked to respond to the ink color and ignore the meaning of the word. The ink color and meaning of the word can be either congruent (e.g., “Blue” written in blue), incongruent (e.g., “Red” written in blue), or neutral (e.g., “a string of 4 Xs” written in blue; see, e.g., Henik et al., 2018). The difference in reaction time (RT) between incongruent and neutral stimuli (i.e., the interference effect) is large and reliable, whereas the difference between neutral and congruent stimuli (i.e., the facilitation effect) is small and fragile. Put another way, result patterns regarding the facilitation effect are mixed. Some studies did not find significant RT differences, some observed faster RTs for congruent than neutral trials, and some studies found faster RTs for neutral compared with congruent trials (Henik et al., 2018; MacLeod, 1991).

It has been suggested that the Stroop task consists of two main conflicts: the information conflict, which arises in incongruent stimuli when word meaning and ink color do not match; and the task conflict, which arises due to the fact that in some cases, stimuli activate task sets that are associated with them (Kiesel et al., 2007; Meiran & Kessler, 2008; Rogers & Monsell, 1995; Waszak et al., 2003; Wendt et al., 2013). Words tend to automatically evoke the task set “reading” (Monsell et al., 2001) and as a result, when one has to name the color of the ink, the task set “word reading” competes with the task set “color naming” and creates a task conflict—a conflict between the automatic task set of reading and the instructed task to name the ink color. Information conflict is commonly assessed by the comparison between incongruent and congruent trials, and task conflict is commonly assessed by the comparison between congruent and neutral trials. While RT measures do not reliably show task conflict—that is, faster RTs for neutral compared with congruent stimuli, there is converging evidence for the assumption of task conflict in neuroimaging studies, RT studies that used specific experimental manipulations, and in recent studies applying pupillometry.

Neuroimaging studies show increased brain activation in incongruent trials compared with congruent trials and to congruent trials compared with neutral trials. Such activations appear in areas that are attributed to conflict monitoring (such as the anterior cingulate cortex; Bench et al., 1993; Carter et al., 1995; Roelofs et al., 2006). These findings correspond with the notion of the existence of both information and task conflicts. The former is reflected in larger activation in incongruent compared with congruent trials, and the latter in larger activation in congruent compared with neutral trials.

Several experimental manipulations were helpful in measuring task conflict reliably. For example, recent studies showed that a decrease in cognitive control might lead to larger task conflict. Specifically, the task conflict can be magnified by using a high number of neutral trials (Entel et al., 2015), deficient control in a stop-signal task (Kalanthroff et al., 2013), changes in the expectation for conflict (Goldfarb & Henik, 2007), and by reducing the preparation time (i.e., the cue–target interval) in a task-switching situation (Kalanthroff & Henik, 2014). In addition, process-dissociation models can account for behavioral results by assuming the existence of both word reading and color naming processes (Klauer et al., 2015).

## Pupillometry and the Stroop task

Common behavioral measures are not always the most sensitive indicators of cognitive processes. It has already been suggested that pupil size might be more sensitive to indicate differences in cognitive processing. For example, changes in pupil size (or, in short, pupillometry) can be used as an indicator of task difficulty (Kahneman & Beatty, 1966). Several studies that examined changes in pupil dilation during Stroop tasks showed an increased pupil diameter during incongruent trials compared with both neutral and congruent trials (Brown et al., 1999; Laeng et al., 2011; Siegle et al., 2004, 2008). Interestingly, Hershman and Henik (2019) found that in addition to the general pattern of larger dilation in incongruent trials compared with both congruent and neutral trials, there were differences between congruent and neutral trials when the stimuli for the neutral trials consisted of letter strings. Specifically, congruent trials led to larger pupil dilation compared with neutral trials. This reverse facilitation (RF) is frequently used as a marker for task conflict (Entel et al., 2015). Hence, RT differences between congruent and neutral conditions seem less sensitive (or less robust) than pupillometric differences (Hershman & Henik, 2019, 2020; Hershman et al., 2020, 2021; Kalanthroff et al., 2013; Kalanthroff & Henik, 2014).

In a study conducted by Banich et al. (2000), the researchers aimed to examine the contribution of automatic activation of an irrelevant task (or an irrelevant attentional set) even when no responses are required. In a functional magnetic resonance imaging (fMRI) study, participants saw word stimuli that were colored in one of three colors (either blue, brown, and yellow, or red, orange, and green). The authors asked the participants to count the number of trials they saw a purple ink color (that actually never occurred) while varying whether the irrelevant word was an incongruent Stroop stimulus or a neutral word stimulus (i.e., a color-unrelated word such as “notion,” “chain,” or “lost”). Nonetheless, without any responses, the experimenters observed greater prefrontal cortex activity in blocks with a 50–50 mix of incongruent and neutral trials than in blocks with 100% neutral trials. The authors interpreted their data as showing that it is harder to maintain an attentional set for color in incongruent compared with neutral trials. However, the comparison between incongruent and neutral trials (that is frequently defined as interference effect) includes both task and information conflict. Thus, we do not know whether the observed differences in the mixed blocks compared with the neutral blocks reflect the effort to maintain the task (attentional) set or are related to information conflict. Moreover, Banich et al. used real words as neutral stimuli. Neutral words induce task conflict (but no information conflict), whereas nonword neutrals (e.g., series of *X*s) may induce task conflict to a much lesser degree (if at all; Hershman et al., 2020, 2021; Monsell et al., 2001). Accordingly, we conjecture it is more likely that Banich et al.’s difference between incongruent and neutral words indicates information conflict only or both information and task conflict. In the present study, we aimed to examine whether task conflict could also appear when no actual response is required, regardless of the information conflict.

## The current study

In the current study, we conducted a color-word Stroop task (Henik et al., 2018) and used RTs and changes in pupil size as dependent measures. We aimed to explore the contribution of the existence of an actual response to both task and information conflicts. For this aim, we conducted an experiment using a novel Stroop design. In this design, similar to the typically used Stroop task, participants were presented with Stroop stimuli and were asked to respond to the ink color. However, in addition to the standard stimuli, the current design included trials that required no response. We call the no response trials “Rest trials.” In our experiment, Rest trials were defined as Stroop stimuli in colors that were not relevant to the task. It means that both Rest and Response trials were congruent, neutral, or incongruent, but we assigned response keys only to two colors out of four possible colors that were presented. In other words, although all trials were Stroop stimuli, there were trials in which no response was required. It is important to note that our Resting Stroop design is unique compared with other inhibition tasks (e.g., the stop-signal task) in the sense that the participants do not need to directly inhibit their responses—there are no possible keys to respond with in the case of the Rest trials.

Measuring pupil changes allowed us to examine both response and no response (i.e., Rest) conditions. The response conditions should present the commonly found information and task conflict. Clearly, our main interest are the Rest trials. If responding is essential to produce conflicts, no indications for conflict should appear in the Rest trials. However, this suggestion (no conflict) might apply to the two conflicts or to only one of them. Moreover, if the two conflicts are independent, it is possible that the lack of response might affect one of them and not the other. In principle, there is no reason to think that task conflict would appear when execution of no task is required. Hence, the view suggesting that task conflict ensues as a reaction to the need to perform task(s), predicts no task conflict on rest trials. In contrast, the view suggesting that task conflict is not dependent on the need to execute task(s), predicts the appearance of task conflict on rest trials. The current design required participants to note the color (in order to decide whether they should respond or not). This, in turn, may lead to task conflict, whether the specific color requires responding or not. In contrast, only the need to respond might create information conflict. Accordingly, analysis of changes in pupil size of Response trials is expected to present both task and information conflict, whereas Rest trials may produce task conflict only. Moreover, analysis of changes in pupil size of Rest trials might provide us with evidence of the contribution of an actual response to these conflicts.

### Materials and methods

#### Participants

Thirty-eight undergraduate students (28 females, mean age 23.21 years old, *SD* = 1.19) from Ben-Gurion University of the Negev participated in the experiment in return for partial fulfillment of course requirements or credit. The sample size was based on a modulation of the sample size from Hershman and Henik (2019) in which RT and pupil dilation measures were collected in a Stroop task. Hershman and Henik used 19 participants, but after taking into consideration dropout rates and a smaller effect size due to the addition of more conditions, we increased our sample size in the current study to 38 participants. The study was approved by the university’s behavioral ethics committee. All participants signed an informed consent form prior to their participation in the experiment. All participants had normal vision without wearing glasses or contact lenses and no reported history of attention deficit disorder or any learning disabilities.

#### Stimuli

Each stimulus consisted of one of four color words—כחול (Hebrew word for blue), אדום (Hebrew word for red), ירוק (Hebrew word for green), צהוב (Hebrew word for yellow)—or a single four-letter string in Hebrew שששש (meaningless repetition of a Hebrew letter, equivalent to XXXX in the English version). The four letters subtended a visual angle of 3.22° to 4.43° for height and 8.49° to 16.24° for width from a viewing distance of about 50 cm. The stimuli were printed in 150-point boldfaced Arial font. The ink color was either red (RGB: 255, 0, 0), blue (RGB: 0, 0, 255), green (RGB: 0, 130, 0) or yellow (RGB: 255, 255, 0). We divided the four colors into two groups: those for Response trials and those for Rest trials. The congruency conditions (congruent, incongruent, and neutral) were built separately for two colors in the Response trials and two colors in the Rest trials so that for each congruency condition, there were six Stroop stimuli—two for each response condition of congruent color words, incongruent color words, and for neutrals (XXXX). The mean luminance for the congruency conditions were 191.5, 191.15, and 191.49 for the congruent, the neutral, and the incongruent stimuli, respectively. The stimuli were presented at the center of a screen on a silver background (RGB: 192, 192, 192; mean luminance = 192). The stimuli were selected randomly.

#### Procedure

The experiment was conducted in a dimly illuminated room. A keyboard was placed on a table between the participant and the monitor. Participants were tested individually. The experimental part included 10 Stroop practice trials (which were not analyzed). If more than one trial was not followed by a correct response, another practice block of 10 trials was presented until the participant had a response rate of at least 90% correct responses. During practice, participants received feedback on accuracy.

The Stroop practice block(s) was followed by the experimental part, which included 624 trials divided into four blocks of 156 experimental trials each. In total, there were 312 Rest trials and 312 Response trials—104 trials for each congruency condition (i.e., congruent, incongruent, and neutral). Words and colors for Rest and Response trials were not intermixed (e.g., if the colors red and blue were used on Response trials and green and yellow were used on Rest trials, then the words RED and BLUE were never presented on Rest trials and the colors green and yellow were never presented on Response trials, and vice versa).

Each trial started with a 1,000-ms fixation (a black “+” sign in the center of the screen), followed by a Stroop stimulus. Half of the participants had to respond to blue and red, and the other half to green and yellow. The mapping of the color (red/blue or yellow/green) to the response key (“b” or “m”) was chosen randomly for each participant. They were instructed to respond only to these colors and to do nothing if the presented stimulus was any other color. All participants were asked to ignore the meaning of the word and to press the correct key as fast as possible without making mistakes. The visual stimulus stayed in view for 400 ms and was followed by a blank screen until a key press or for a maximum of 1,100 ms. RT was calculated from the appearance of the visual stimulus to the onset of response. Each trial ended with a 1,500-ms intertrial interval.

#### Apparatus

Pupil size was measured using a video-based desktop-mounted eye tracker (The Eye Tribe) with a sampling rate of 60 Hz (16.66 ms intersampling time). Stimulus presentation and data acquisition were controlled by Psychtoolbox software (Version 3.0.14) on MATLAB (The MathWorks Version 9.4.0.813654 [R2018a]). Stimuli were displayed on a 23-inch LED monitor (Dell E2314Hf) at a resolution of 1,920 × 1,080 pixels, with a refresh rate of 60 Hz. The participant’s head was positioned on a chin rest, and the distance from the eyes to the monitor was set at about 50 cm. To maintain an accurate measurement of pupil size during the task, participants were required to keep their eyes fixated on the center of the screen and to avoid eye movements for the entire task. The pupil area was determined using the Eye Tribe algorithm.

### Results

#### Preprocessing

We excluded data of six participants from the analysis who did not have at least 70 valid trials (correct responses with no more than 30% of missing pupillometric values) in each condition. After the exclusion, we had 32 participants (23 females, mean age = 23.37 years old, *SD* = 1.18). Pupil data were processed using the CHAP software (Hershman et al., 2019). First, pupil data (that were measured during the whole experiment) were extracted from the Eye Tribe (pupil size in arbitrary units). For each experimental trial, we used pupillometric values in the time window of 2,300 ms starting 500 ms before stimulus onset (and lasting 1,800 ms after stimulus onset). Then, we removed outlier samples with *Z*-scores larger than 2.5. Z-scores were calculated based on the mean and standard deviation calculated for the pupil dilation measure for each trial. Next, we calculated the percent of outlier measures for each participant in each trial and excluded from analysis trials with more than 30% of missing values. We also excluded trials with incorrect or missing responses. We defined a minimum number of 70 valid trials for each condition so that if removing outlier trials resulted in less than a total of 70 trials per condition, the participant was excluded from the analysis. Next, we detected eye blinks by using Hershman et al.’s (2018) algorithm and filled missing values by using linear interpolation (Hershman & Henik, 2019). Then, time courses were aligned with the onset of the Stroop stimulus and divided by the baseline (baseline was defined as the average pupil size 500 ms before the stimulus onset). This preprocessing of pupil data eliminated 7.01% of trials on average. The exclusion rate in each condition is presented in Table 1.

**Table 1:** Exclusion rate in each condition in the experiment

	Congruent	Incongruent	Neutral
Response	8.74%	11.39%	9.7%
Rest	4.57%	4.3%	3.36%

We excluded incorrect trials and trials with more than 30% of missing values from the analysis

#### Reaction time

**Fig. 1:**
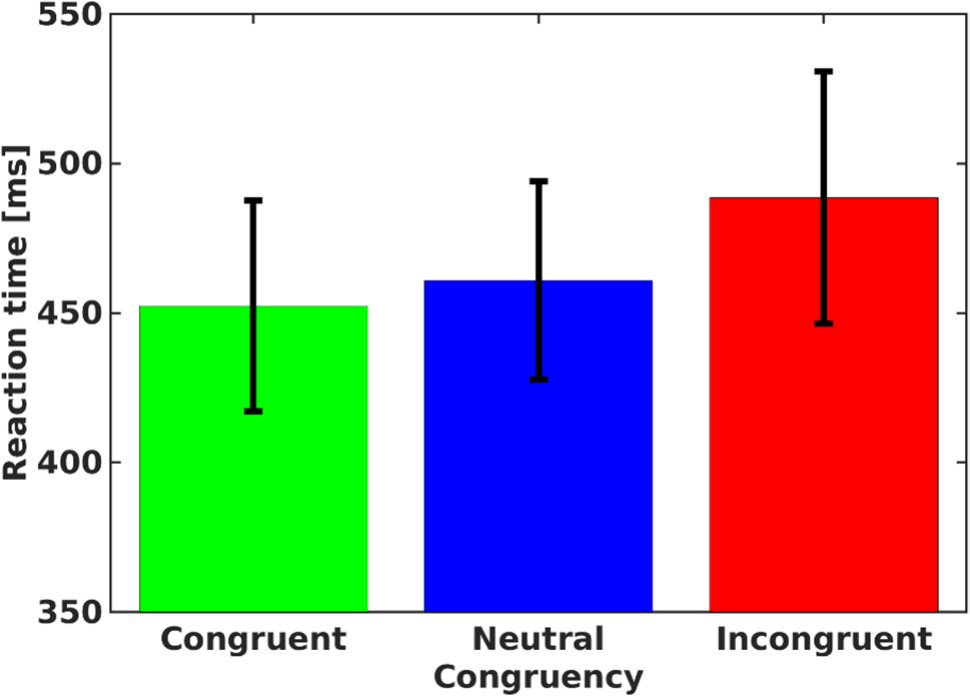
Mean reaction time for each congruency condition of the response trials. *Note.* Error bars represent a 95% confidence interval from the mean. (Color figure online)

Mean RTs (mean RTs in the various conditions are presented in Fig. 1) of correct (pupil valid) trials (trials with no more than 20% of missing samples) for each participant in each condition of the Response trials were subjected to a one-way repeated-measures analysis of variance (ANOVA), with congruency (congruent, neutral, incongruent) as an independent factor. As expected, an omnibus analysis produced a significant congruency effect, $F\left(2,62\right)=23.172,p<.001,{\upeta}_{\textrm{p}}^2=.428,B{F}_{10}>{10}^5$. Single contrasts indicated strong evidence for information conflict (mean RT was longer in incongruent compared with congruent trials), *F*(1, 31) = 47.81, *p* < .001, *BF*_10_ > 10^5^, and interference (mean RT was longer in incongruent compared with neutral trials), *F*(1, 31) = 16.28, *p* < .001, *BF*_10_ = 87. No differences were found between neutral and congruent trials, *F*(1, 31) = 3.97, *p* = .055, *BF*_10_ = 1.08.

#### Pupil dilation

In order to examine the temporal differences among the conditions, we used the approach of Hershman and Henik (2019). Specifically, we ran time-series analyses in terms of Bayesian paired-sample *t* tests between the conditions of interest. First, we compared pupil dilation data for all Rest to all Response trials. The Rest trials showed less dilation compared with Response trials in most parts of the time window. Specifically, the difference between Rest and Response trials started after about 500 ms poststimulus onset and stayed until the end of the trial. These results suggested that the Rest trials required less mental effort in terms of cognitive control.

#### Analysis of response trials

**Fig. 2:**
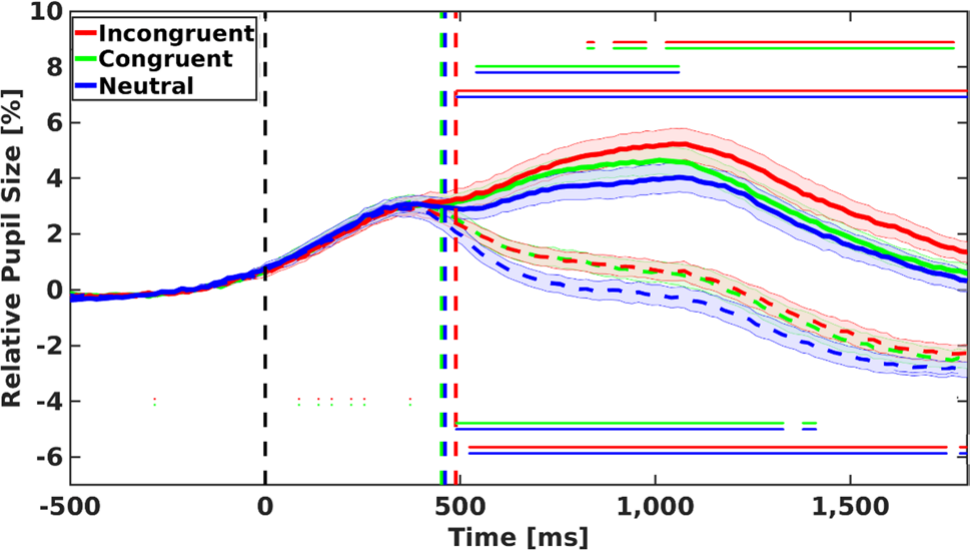
Mean relative pupil dilation (compared with the average of 500 ms before the stimulus onset) for both response (solid lines) and rest trials (dashed lines). *Note.* Each curve presents changes in pupil dilation as a function of time. The shaded areas represent one standard error from the mean. The solid lines represent Response trials, and the dashed lines represent Rest trials. The horizontal double-lines indicate meaningful differences (i.e., *BF*_10_ ≥ 3) between conditions. The upper horizontal double-lines indicate meaningful differences between Response trials, and the lower horizontal double-lines indicate meaningful differences between Rest trials. (Color figure online)

In line with previous studies (Hershman et al., 2020, 2021; Hershman & Henik, 2019, 2020), we investigated the results of pupil dilation in the Stroop task. In order to ensure that pupil dilation patterns replicated previous findings, we analyzed the Response trials to assess whether there were meaningful differences (i.e., *BF*_10_ > 3) among all the investigated conditions. Our analysis (see Fig. 2) indicates meaningful evidence for task conflict (i.e., larger dilation in congruent (solid green) trials than in neutral (solid blue) trials). The differences between the conditions appeared at about 540 ms poststimulus onset and stayed until about 1,060 ms post-stimulus onset. In addition, we observed meaningful evidence for information conflict (i.e., larger dilation in incongruent (solid red) trials than in congruent (solid green) trials). The differences between the conditions appeared after about 890 ms and stayed until the end of the trial. Moreover, our analysis indicates meaningful differences between the incongruent (solid red) and neutral (solid blue) trials. These differences appeared after about 490 ms post-stimulus onset and stayed until the end of the trial. These results are in line with previous pupillometric studies (Hershman et al., 2020, 2021; Hershman & Henik, 2019, 2020) that suggest that the Stroop task includes two conflicts: information conflict (i.e., a contradiction between word meaning and ink color in incongruent stimuli) and task conflict (i.e., word reading competes with color naming). As can also be seen in Fig. 2, the congruent and incongruent lines are inseparable early on, whereas the line describing the neutral trials starts diverging from these two (i.e., congruent and incongruent) early on (after about 540 ms poststimulus onset). In contrast, the congruent and incongruent trials start diverging later. This pattern is in line with recent studies suggesting that task conflict appears before the information conflict (Goldfarb & Henik, 2007; Hershman et al., 2020, 2021; Hershman & Henik, 2019, 2020; Kalanthroff et al., 2018).

#### Analysis of rest trials

Mean relative changes of the pupil size of correct (pupil valid) trials in each condition of the Rest trials are also presented in Fig. 2 with dashed curves. Our analysis indicates meaningful evidence for task conflict (i.e., larger dilation in congruent (dashed green) trials than in neutral (dashed blue) trials). The differences between the conditions appeared at about 490 ms after the stimulus onset—similar to the general pattern found in the Response trials. These differences stayed until 1,330 ms poststimulus onset. In addition, our analysis indicates meaningful differences between incongruent (dashed red) and neutral (dashed blue) trials. These differences appeared after about 520 ms poststimulus onset and stayed until the end of the trial. In contrast to Response trials, no evidence for information conflict (i.e., larger dilation in incongruent trials than congruent trials) was found.

### Discussion

In the present study, we used Stroop stimuli for both Rest and Response trials. Consequently, the Rest trials differed from the Response trials only because the Rest trials did not have designated keys for a response. As a result, we had two types of stimuli that differed only regarding responding. The analysis of the Response trials led to the same conclusions as in previous Stroop and pupillometry studies (Hershman et al., 2020, 2021; Hershman & Henik, 2019, 2020). Specifically, RT showed evidence of information conflict but not task conflict. In contrast, analysis of the changes of pupil size provided evidence for both task and information conflicts. Moreover, the analysis provided evidence for the temporal priority of the task conflict compared with the information conflict. These findings are in line with the results of previous Stroop studies (Goldfarb & Henik, 2007; Hershman et al., 2020, 2021; Hershman & Henik, 2019, 2020; Kalanthroff et al., 2018).

In addition to the analysis of the Response trials, we also analyzed the Rest trials. While it is not possible to analyze the RTs of these trials (because there were no motor responses), the pupils provided information about the mental effort in the absence of motor response. The analysis of the Rest trials provided evidence for the existence of task conflict even when no response was required. However, no evidence of information conflict was found. These results might suggest that information conflict is dependent on a direct requirement for a response (or at least with an association between the stimuli to an actual response). In contrast, the task conflict arises from exposure to stimuli and the task set to respond to some of them, but task conflict does not require a motor response.

We have considerable reason to assume that (in common Stroop task studies; i.e., in response trials) congruent stimuli elicit competition in the task sets of color identification and word reading (Littman et al., 2019). This is because congruent trials have been associated with more brain activity than neutral trials in areas assumed to be related to conflict monitoring or conflict resolution, and some Stroop studies even yielded worse performance in congruent trials than in neutral trials (Bench et al., 1993; Carter et al., 1995; Roelofs et al., 2006). The fact that performance is often not worse in congruent than in neutral trials can be explained by assuming that task conflict is (present but) masked by facilitation due to the congruent word meaning which facilitates response selection/execution (Goldfarb & Henik, 2007). Stroop studies applying pupillometry, consistently demonstrated larger pupil dilation for congruent than for neutral trials—even in the absence of a corresponding performance difference (Hershman et al., 2020, 2021, 2022; Hershman & Henik, 2019, 2020). Pupil dilation in Rest trials is also larger for congruent than for neutral trials. Moreover, this effect occurs in a similar time window as in response trials. Therefore, we conjecture that larger pupil dilation in congruent Rest trials than in neutral Rest trials reflects task conflict.

These findings are in line with Banich et al.’s (2000) findings that showed greater prefrontal cortex activity for blocks with both neutral (word) and incongruent Stroop trials than in blocks with only neutral trials. However, the conclusions regarding task conflict reached in this and in our study are based on different data; the difference between partially incongruent and only neutral (i.e., interference) blocks in the Banich et al. study and the difference between congruent and neutral (i.e., reverse facilitation) trials in the current study. Moreover, the neutral trials in the two studies were different; words in the Banich et al. study and series of *X*s in the current study. Neutral words induce task conflict but no information conflict, whereas nonword neutrals (e.g., series of *X*s) may induce task conflict to a much lesser degree (if at all). Accordingly, Banich et al.’s difference between incongruent and neural words might indicate information conflict only or both information and task conflict. It is not clear whether Banich et al.’s comparison between incongruent and neutral words can be taken as evidence for the existence of task conflict. In contrast, the reverse facilitation (i.e., the difference between nonword neutral and congruent trials), found in the current study constitutes evidence for task conflict not contaminated by information conflict. Please note that when this indication (i.e., reverse facilitation) of task conflict appears, there is no indication of information conflict at all; as indicated by Bayes factors below 1/3 for the comparison of the conditions in the time window between about 540 ms post the stimulus onset until the end of the trial.

Incongruent Rest trials (as well as incongruent Response trials) involved semantic conflict (i.e., competition between two pieces of contradicting semantic properties), yet incongruent Rest trials do not involve response conflict (i.e., competition between motor responses). Hershman and Henik (2020) used De Houwer’s (2003) 2:1 paradigm to examine changes in pupil size for each component of the information conflict (i.e., semantic and response conflicts). The results (evidence for both semantic and response conflicts) suggested that pupil dilation might be used as an indicator for both semantic and response conflicts. Hence, the absence of information conflict (in terms of changes in pupil size) suggests that the absence of an actual requirement to respond to the Rest stimuli caused less information conflict (i.e., semantic and response conflicts).

The Stroop task (Stroop, 1935) shows that the meaning of a word modulates responding to the color. Importantly, the Stroop task always requires a motor response to the color. In the present study, the Rest trials feature no response to the color. It is conceivable that, in this case, no task conflict would emerge. Because there is no need to respond, there is no need to decide what task to prefer. Hence, one might assume that no task conflict arises. In contrast with the “no-task conflict on Rest trials” conjecture, we found task conflict when no response was required. Theories of the Stroop task (and possibly Stroop-like tasks) have to take this into account. Needless to say, it is possible that the need to respond on the response trials does affect the Rest trials and triggers task conflict even when no response is required. This possibility, as well as other aspects of task conflict when no response is required, might be examined in further studies.

One can argue that the observed reverse facilitation (smaller pupil dilation for neutral trials than congruent trials) can be explained by a source unrelated to conflict due to “sensitivity to perceivable letter strings.”^1^ Further, one may assume that a larger pupil response may accompany a word than letter strings because it is more arousing. Previous Stroop and pupillometry studies examined different kinds of neutrals (Hershman et al., 2020, 2021). Specifically, in these studies, the observed reverse facilitation was replicated when the neutral stimuli were colored patches, symbols, pseudowords (i.e., meaningless words), and also abstract draws and colored patches. Consistently, these findings suggested that the more meaningless a stimulus is (in terms of semantic/phonological/orthographical meaning), the less task conflict will be observed (Hershman et al., 2021). Therefore, both arousal and “sensitivity to perceivable letter strings” cannot explain the observed well-replicated findings. Indeed, pseudowords might be used as neutrals instead of letter strings, but it has already been suggested that pseudowords (which elicit less task conflict compared with congruent and incongruent trials) might result in larger task conflict compared with letter strings. In the present study, we chose meaningless neutral stimuli (i.e., letter strings) to decrease the task conflict in the neutral condition.

Another interesting explanation for our findings could be contingent attentional capture (Folk et al., 1992). Such capture might lead to greater pupil diameter in both congruent and incongruent trials than in neutral in both Response and Rest trials. However, in a recent study (Hershman et al., 2020), pseudowords (as well as letter strings and symbols) were compared with real words and both congruent and incongruent trials. Pseudowords have the same orthographical and phonological features as real words. Accordingly, responding (RT and pupil size) to pseudowords should be similar to responding to words. However, responding to pseudowords was different than responding to word stimuli. Pseudowords showed smaller task conflict than all word stimuli. Specifically, reverse facilitation was found for pseudowords but not for real words. In this study (Hershman et al., 2020), the evidence for task conflict (i.e., reverse facilitation) was found only with pupillometry and not with RTs, which suggested that all the examined neutrals required the same mental effort. Therefore, we conjecture contingent attentional capture is a less likely explanation for our findings.

Please note that task conflict has been conceptualized in different ways. One parsimonious concept refers to some kind of resource limitations (current theorizing would assume working memory limitations, previously, this was often conceptualized as attentional limitations) when performing two tasks (like color naming and automatic word reading) simultaneously (see, e.g., Koch et al., 2018, for a recent overview). As such, “being engaged in two tasks at the same time” is sufficient to induce task conflict even if one task like reading is not instructed but performed habitually. We are aware that this is the “minimum” concept of task conflict. If tasks overlap in terms of stimulus and response sets, more processes in terms of attentional selection and/or inhibition of response sets might add to task conflict. In a further study that was done in our lab (Hershman et al., 2023), we aimed to investigate the contribution of task-switching to task conflict. Participants were asked to respond to the color or to the meaning of the stimuli according to a randomly varying task cue (e.g., a circle for color naming and a square for word reading presented around the stimuli). Our results revealed evidence for task conflict in task switch trials. In contrast, in task repetition trials, no evidence for task conflict was found. We interpreted this pattern as indicative of different levels of control in terms of task-set implementation, which is stronger in repetition trials than in switch trials.

In general, our results suggest that conflict resolution should be explored and dissociated from the requirement to respond and that using an RT measure that is dependent on response can affect the pattern of results and the models that stem from it. Given the fact that most studies and cognitive control models (e.g., the proactive control/task conflict [PC-TC] model of Kalanthroff et al., 2018) are based on experiments in which a response is made, it is important to explore the possibility that such models do not accurately reflect cognitive processing in tasks where a response is not required. Future studies should explore further the possibility of combining measures such as pupil size in addition to the common RT and explore different experimental designs in which response and conflict are dissociated.

## Footnotes

1 We thank an anonymous reviewer for pointing out this alternative.
